# Omnidirectional acoustic cloaking against airborne sound realized by a locally resonant sonic material

**DOI:** 10.1038/s41598-022-20591-z

**Published:** 2022-09-30

**Authors:** Kei Matsushima, Yuki Noguchi, Takayuki Yamada

**Affiliations:** 1 Department of Mechanical Engineering, Graduate School of Engineering, The University of Tokyo, Tokyo, 113-8656 Japan; 2 Department of Strategic Studies, Institute of Engineering Innovation, Graduate School of Engineering, The University of Tokyo, Tokyo, 113-8656 Japan

**Keywords:** Acoustics, Mechanical engineering

## Abstract

We report that a locally resonant sonic material realizes omnidirectional acoustic invisibility in air. To achieve acoustic cloaking in the low-frequency regime, we axisymmetrically placed elastic rods comprised of silicone rubber and lead around a cloaked object. The radii of the rods are designed to minimize their total scattering cross section for a given frequency. The optimization is performed using an algorithm incorporating multiple scattering theory and gradient-based nonlinear programming. We numerically confirmed that the designed cloaking device suppressed the scattering cross section by almost 92% for all incident directions at the target frequency.

## Introduction

Invisibility cloaking remains one of the most exotic phenomena in optics and acoustics. The primary concept behind invisibility cloaking is to cover a scatterer with carefully designed objects such that they suppress or cancel the scattering.

After Pendry et al.^1^ reported that a coordinate-transformation technique can determine a material parameter distribution for perfect cloaking, many studies have been devoted to theoretical, numerical, and experimental realization of this transformation-based cloaking in acoustics^2–10^. Because the transformation optics and acoustics require a continuously inhomogeneous and anisotropic material with extreme values, its experimental realization is not easy. The scattering-cancellation technique is another approach to realize optical and acoustic cloaking^11–18^. While the scattering-cancellation approach does not require the continuous and anisotropic material distribution, higher and lower mass densities than the background medium are still required, which is very difficult for airborne sound cloaking.

For acoustic invisibility, a more feasible approach is to design a cloaking object’s geometry rather than manipulate its material properties. For example, Garcia-Chocano et al.^19^ reported that aluminum rods distributed around a cylindrical object can significantly suppress its acoustic scattering by optimizing the positions of the rods. This geometry technique is not limited to cylindrical objects, but more complex geometries can be designed by optimization techniques^20–27^. However, the main drawback of geometry-based approaches is that designed devices often lack cylindrical symmetry, i.e., invisibility cloaking only works for specific incident directions.

This challenge was recently tackled by Jo et al.^28^ by arranging axisymmetric cylindrical lattices around a core object and optimizing their radii and positions to suppress acoustic scattering. Because they used a rigid material for the cloaking device, the cloaking effect originates from the Bragg scattering among the lattice. This implies that the size of the device must be several times larger than the target wavelength.

In this report, we demonstrate a locally resonant sonic material that realizes omnidirectional acoustic cloaking from airborne sound in the low-frequency regime. Our cloaked object model is similar to those proposed by Garcia-Chocano et al.^19^ and Jo et al.^28^, i.e., a rigid circular object with surrounding elastic rods. We used silicone rubber and lead to construct the surrounding rods in order to induce local resonance in the low-frequency range^29^. We fixed the positions of the rods and optimized their radii to suppress the total scattering cross section for a given frequency. The calculation is performed using multiple scattering theory^30–32^. After the optimization, we observed that the designed cloaking device reduces the scattering cross section by almost $92\%$ for every direction.

## Model and formulation

**Figure 1:**
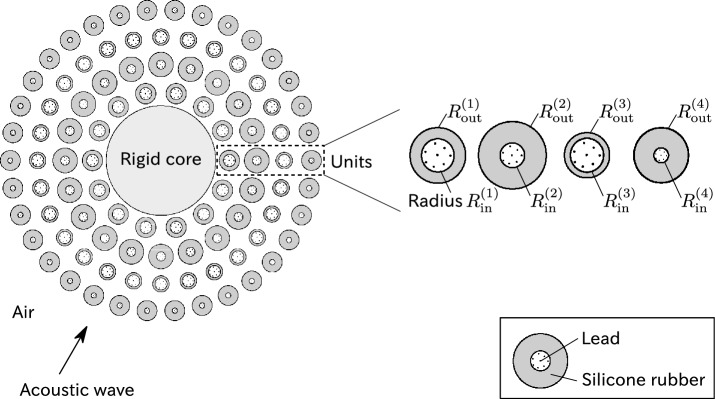
Rigid-material core covered with rubber-lead rods. The number of rods in each layer, from innermost to outermost, are 14, 20, 28, and 34. The materials are placed in air and illuminated by an incident acoustic wave. The radii $R_{\mathrm {out}}^{(1)},R_{\mathrm {in}}^{(1)},R_{\mathrm {out}}^{(2)},R_{\mathrm {in}}^{(2)},R_{\mathrm {out}}^{(3)},R_{\mathrm {in}}^{(3)},R_{\mathrm {out}}^{(4)},R_{\mathrm {in}}^{(4)}$ are optimized by the proposed method. The core is placed at the origin. The centers of the rods are located at (1.25*R*, 0), (1.75*R*, 0), (2.25*R*, 0), and (2.75*R*, 0) (from innermost to outermost), where *R* is the radius of the core.

We define the model to achieve omnidirectional acoustic cloaking of a cylindrical object in air (mass density $\rho = 1.2\,{\mathrm {kg}}/{\mathrm {m}}^3$ and bulk modulus $\kappa = 1.4\times 10^5\,{\mathrm {Pa}}$). As shown in Fig. 1, we consider a rigid-material cylindrical core surrounded by $N=96$ rods. Each rod is comprised of silicone rubber^33^ (mass density $1.3\times 10^3\,{\mathrm {kg}}/{\mathrm {m}}^3$, bulk modulus $6.3\times 10^5\,{\mathrm {Pa}}$, and shear modulus $4.0\times 10^4\,{\mathrm {Pa}}$) encapsulating lead^34^ (mass density $11.6\times 10^3\,{\mathrm {kg}}/{\mathrm {m}}^3$, bulk modulus $5.22\times 10^{10}\,{\mathrm {Pa}}$, and shear modulus $1.49\times 10^{10}\,{\mathrm {Pa}}$) placed at the center.

For a given operating frequency, we optimized the radii of each rubber-lead rod to minimize their total scattering cross section. The cross section is computed using multiple scattering theory. To this end, we assume that sound pressure *p* is governed by the Helmholtz equation in air, i.e.,

1 $$\begin{aligned} \frac{1}{\rho }\nabla ^2 p(x) + \frac{\omega ^2}{\kappa } p(x) = 0, \end{aligned}$$

where $\omega >0$ is a given angular frequency. Since the silicone rubber and lead have non-negligible shear moduli, we solve Navier’s equation

2 $$\begin{aligned} \nabla \cdot \sigma (x) + \rho _{\mathrm {e}}(x)\omega ^2 u(x) = 0 \end{aligned}$$

under the plane-strain condition to obtain the displacement *u* and stress $\sigma$ in the elastic rods, where $\rho _{\mathrm {e}}$ is the mass density in solid. The partial differential equations are coupled by the following interface conditions:

3 $$\begin{aligned} \sigma ^T n = -pn, \end{aligned}$$

4 $$\begin{aligned} \nabla p\cdot n = \rho \omega ^2 u\cdot n, \end{aligned}$$

where *n* is the unit outward normal vector.

Acoustic cloaking is achieved by minimizing the scattering cross section $\sigma _{\mathrm {cloaked}}$. To accomplish this, we set the design parameters as the radii of the rods aligned along each layer as shown in Fig. 1. To ensure that the rods are well-separated, we also impose the constraints

5 $$\begin{aligned} 0 \le R_{\mathrm {in}}^{(i)}< R_{\mathrm {out}}^{(i)} < 0.25 R, \end{aligned}$$

where *R* is the radius of the core. The nonlinear optimization problem with linear constraints is solved by sequential least-squares quadratic programming^35, 36^, implemented in NLopt^37^.

## Results

### Designed cloaking device

**Table 1:** Optimized radii of the rubber-lead rods.

*i*	1	2	3	4
$R_{\mathrm {out}}^{(i)}/R$	0.19909	0.23752	0.16915	0.16365
$R_{\mathrm {in}}^{(i)}/R$	0.02452	0.09350	0.00797	0.00000

In Table 1, we list the optimized radii of the rubber-lead rods when we gave a plane wave propagating in the positive $x_2$ direction with normalized frequency $\omega R/(2\pi c)=0.15915$, where *c* is the speed of sound in air. The minimized objective value is $\sigma _{\mathrm {cloaked}}/\sigma _{\mathrm {bare}} = 7.5842\times 10^{-2}$, where $\sigma _{\mathrm {bare}}$ is the scattering cross section of the bare core at the same frequency. From the results, we observed that the designed cloak suppresses scattering by almost 92%.

**Figure 2:**
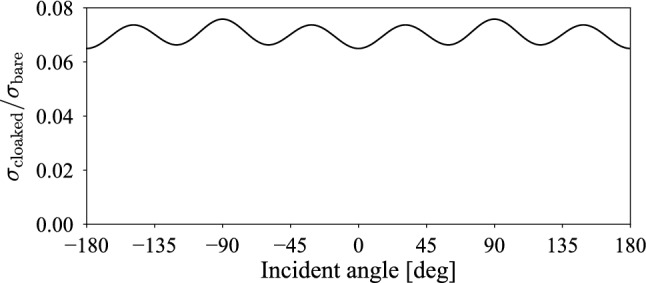
Scattering cross section of the designed cloak at various incident angles.

To confirm that the designed cloak is omnidirectional, we varied the direction of the incident plane wave and plotted the corresponding scattering cross section in Fig. 2. From the results, we note that the scattering cross section $\sigma _{\mathrm {cloaked}}/\sigma _{\mathrm {bare}}$ is only minorly sensitive to incident angle, and all the values are far less than unity. This implies that the designed cloak works for any incident angle, thus achieving omnidirectional cloaking.

**Figure 3:**
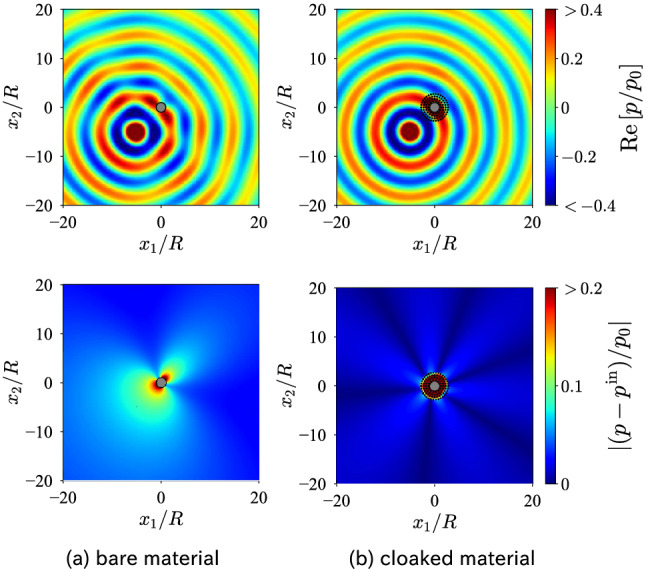
Acoustic field *p* and scattered field $p-p^{\mathrm {in}}$ when a monopole source $p^{\mathrm {in}}=p_0 H^{(1)}_0(k|x-x_0|)$ is present. The source is located at $x_0=(-5R,-5R)$. The dots in (b) represent the positions of the rubber-lead rods.

The omnidirectional cloak successfully hides the core object from any time-harmonic waves as they can be written as a superposition of plane waves. We placed a monopole source at $x_0=(-5R,-5R)$ and defined the incident wave as $p_0 H^{(1)}_0(k|x-x_0|)$ instead of the plane wave. Figure 3 illustrates the sound fields for the bare and cloaked objects. From the results, we observe that the forward and backward scattering are significantly suppressed outside the designed layers. The layers localize the incident wave around the core and magnify its amplitude. This implies that high-quality resonance contributes to the cloaking effect.

**Figure 4:**
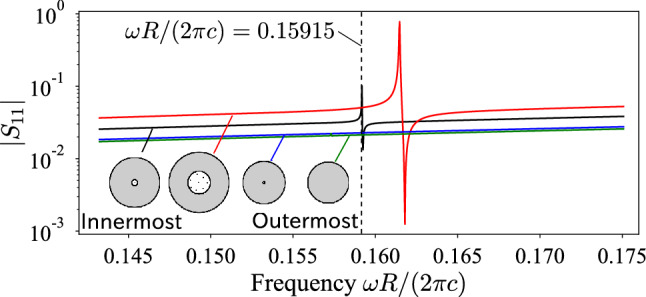
Dipolar component spectrum of the scattering matrix *S* for each rubber-lead rod.

To confirm that the cloaking is associated with the resonance, we calculated the dipolar component $S_{11}$ of each rubber-lead rod’s scattering matrix *S*. The results are shown in Fig. 4. At the target frequency $\omega R/(2\pi c)=0.15915$, the innermost rod exhibits a sharp dipolar resonance whereas its neighbor has a higher resonant frequency. It is well known that a dipolar resonance contributes to a dramatic shift in macroscopic mass density, indicating that the cloaking is achieved by optimizing layer-dependent effective material parameters.

**Figure 5:**
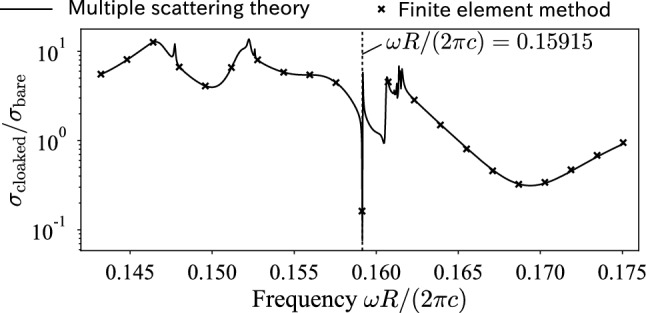
Scattering cross section of the designed cloaked material when the upward-propagating plane wave is given for various frequencies.

We are also interested in how the cloaking performance depends on the operating frequency. In Fig. 5, we plotted the spectrum of the scattering cross section when a plane wave propagates in the positive $x_2$ direction. To check the accuracy of multiple scattering theory, we conducted the same simulation using a mode-matching finite element method^38^, implemented in FreeFem++^39^. Both multiple scattering theory and the finite element method yielded consistent values, verifying the calculations in this study. In the spectrum, we observed a sharp dip at the target frequency, meaning that the cloaking only works in a narrow frequency band. However, the spectrum showed that the scattering cross section is also suppressed in another narrow band $0.165<\omega R/(2\pi c)<0.175$, though this range is not targeted in the optimization process. This narrow-band cloaking also appears to be associated with the dipolar resonance at $\omega R/(2\pi c)=0.162$ in Fig. 4. In the lower frequency range, the cloaked object exhibited larger scattering cross section than the bare one. This is because the resonance effect is no longer available in this spectrum; thus, the designed rods simply increased the geometrical cross section.

### Viscoelasticity

As the proposed structure utilizes the local resonance of rubber-lead cells, we are interested in whether the cloaking effect is valid even when the materials have non-negligible viscosity, which may break the low-frequency local resonance. Acoustic waves lose their energy mainly due to the viscosity of the solid materials and viscothermal boundary layers at the solid-fluid interfaces^40^. To model the material losses, we replace the elastic tensor *C* of the silicone rubber with the complex value $C_0 {\mathrm {e}}^{-{\mathrm {i}}\delta }$, where $C_0$ is the original constant, and $\tan \delta \ge 0$ is the loss tangent.

**Figure 6:**
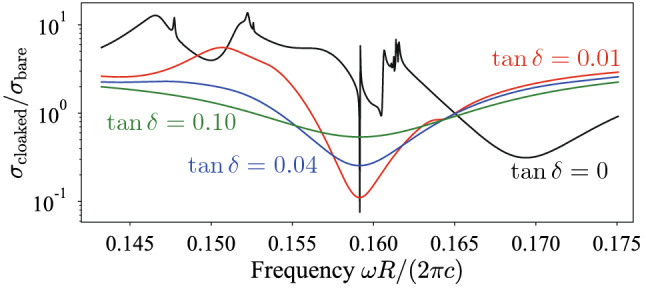
Scattering cross section of the designed cloaked materials with loss tangent $\tan \delta$.

We performed the same optimization for $\tan \delta = 0.01,0.04,0.10$ and list the optimized radii in Table 2. From the results, we observe that even the small viscosity changes the optimized values. In addition, we plot the spectrum of the scattering cross section for the viscoelastic system in Fig. 6. As expected, the introduced viscosity deteriorates the minimum value in the spectrum, while the cloaking bandwidth is slightly broadened. The result indicates that the cloaking effect $\sigma _{\mathrm {cloaked}}/\sigma _{\mathrm {bare}}<1$ is still observed even if a small viscosity is introduced in the system.

**Table 2:** Optimized radii of the rubber-lead rods for various $\tan \delta$.

*i*		1	2	3	4
$\tan \delta =0.00$	$R_{\mathrm {out}}^{(i)}/R$	0.19909	0.23752	0.16915	0.16365
	$R_{\mathrm {in}}^{(i)}/R$	0.02452	0.09350	0.00797	0.00000
$\tan \delta =0.01$	$R_{\mathrm {out}}^{(i)}/R$	0.19207	0.21153	0.19316	0.16445
	$R_{\mathrm {in}}^{(i)}/R$	0.00000	0.00000	0.02379	0.00000
$\tan \delta =0.04$	$R_{\mathrm {out}}^{(i)}/R$	0.18720	0.20698	0.19073	0.16765
	$R_{\mathrm {in}}^{(i)}/R$	0.00000	0.00000	0.03251	0.00000
$\tan \delta =0.10$	$R_{\mathrm {out}}^{(i)}/R$	0.19043	0.22659	0.18315	0.17142
	$R_{\mathrm {in}}^{(i)}/R$	0.00000	0.05137	0.00000	0.00000

## Conclusions

In conclusion, we designed a locally resonant sonic material to realize acoustic cloaking against airborne sound. Multiple scattering theory is formulated to calculate the scattering cross section of multiple elastic rods. The geometry of the elastic rods are optimized such that their cross section is minimized at the target frequency. We successfully demonstrated that the designed cloaking works for every incident direction within a narrow frequency band. Another optimization showed that the proposed cloaking system is still valid even if a small amount of loss is introduced in the solid. Future directions of this work include a comprehensive study of viscothermal losses induced by the boundary-layer effect in air. Another open question is whether the proposed system can realize broadband acoustic cloaking.

## Methods

### Multiple scattering theory

Multiple scattering theory expresses a solution *p* to the coupled scattering problem as

6 $$\begin{aligned} p(x) = \sum _{i=1}^{N+1} \sum _{n=-\infty }^\infty A^{(i)}_n J_n(k|x-c^{(i)}|)\exp ({{\mathrm {i}}n\theta (x-c^{(i)})}) + \sum _{i=1}^{N+1} \sum _{n=-\infty }^\infty B^{(i)}_n H^{(1)}_n(k|x-c^{(i)}|)\exp ({{\mathrm {i}}n\theta (x-c^{(i)})}) , \end{aligned}$$

where $\theta (x)=\tan ^{-1}(x_2/x_1)$ denotes the angle between *x* and the $x_1$ axis, $k=\omega \sqrt{\rho /\kappa }$ is the wavenumber, and $J_n$ is the Bessel function of *n*th order. Under the time-harmonic assumption with ${\mathrm {e}}^{-{\mathrm {i}}\omega t}$, the Hankel functions $H^{(1)}_n$ represent radiating waves from each rods. The coefficients $A^{(i)}_n$ and $B^{(i)}_n$ are associated with the *i*th rod (where index $N+1$ denotes the core), whose center is $c^{(i)}$, and are determined by solving a linear system of equations, called a multiple-scattering equation, of the following form:

7 $$\begin{aligned} (I-ST)B = Sb, \quad B=SA. \end{aligned}$$

The matrix *S* is comprised of scattering matrices of the rubber-lead rods, which depend on the configurations (radii, material parameters, and frequency) of the rods, while the matrix *T* is a function of only the rods’ positions $c^{(i)}$ and frequency. The vector *b* is determined by the incident wave^32^.

### Mode-matching finite element method

The finite element simulation was conducted by the open source software FreeFem++^39^. To solve the exterior coupled problem, FreeFem++ discretizes the variational equation

8 $$\begin{aligned} \int _{\Omega _{\mathrm {air}}} \frac{1}{\rho } \nabla p\cdot \nabla \tilde{p} {\mathrm {d}}\Omega - \omega ^2 \int _{\Omega _{\mathrm {air}}} \frac{1}{\kappa } p \tilde{p} {\mathrm {d}}\Omega - \int _{\partial \Omega _{\mathrm {air}}} \frac{1}{\rho } \frac{\partial p}{\partial n}\tilde{p}{\mathrm {d}}\Gamma = 0, \end{aligned}$$

in air region $\Omega _{\mathrm {air}}$ with elastic counterpart

9 $$\begin{aligned} \int _{\Omega _{\mathrm {rod}}} \varepsilon (\tilde{u}):\sigma {\mathrm {d}}\Omega -\omega ^2 \int _{\Omega _{\mathrm {rod}}} \rho _{\mathrm {e}} u\cdot \tilde{u}{\mathrm {d}}\Omega - \int _{\partial \Omega _{\mathrm {rod}}} \tilde{u}\cdot \sigma n {\mathrm {d}}\Gamma = 0, \end{aligned}$$

for test functions $\tilde{p}$ and $\tilde{u}$, where $\varepsilon (\tilde{u})$ is the strain field induced by the displacement $\tilde{u}$, and $\Omega _{\mathrm {rod}}$ represents the rubber-lead rods surrounding the core. The two formulations are coupled by the interface conditions (3) and (4). To impose the Sommerfeld radiation condition, we truncated the unbounded air region $\Omega _{\mathrm {air}}$ by a fictitious circle $\partial B_{R_{\mathrm {ext}}}$ of radius $R_{\mathrm {ext}}>0$. The radius $R_{\mathrm {ext}}$ is large enough to enclose the rods $\Omega _{\mathrm {rod}}$ and core. Outside the fictitious disk $B_{R_{\mathrm {ext}}}$, we have the following multipole expansion

10 $$\begin{aligned} p(x) = \sum _{n=-\infty }^\infty A^{\mathrm {all}}_n J_n(k|x|)\exp ({{\mathrm {i}}n\theta (x)}) + \sum _{n=-\infty }^\infty B^{\mathrm {all}}_n H^{(1)}_n(k|x|)\exp ({{\mathrm {i}}n\theta (x)}), \end{aligned}$$

with given coefficients $A^{\mathrm {all}}_n$ (incident wave). The unknown coefficients $B^{\mathrm {all}}_n$ are obtained by matching the sound pressure *p* and its normal flux $\frac{\partial p}{\partial n}$ with the interior solution on $\partial B_{R_{\mathrm {ext}}}$. For details, see the reference^38^.

## Data Availability

Fortran codes for the multiple scattering analysis and optimization are available at https://github.com/k-matsushima-19/omnicloak.

## References

[1] Pendry JB, Schurig D, Smith DR (2006). Controlling electromagnetic fields. Science.

[2] Cummer SA, Schurig D (2007). One path to acoustic cloaking. N. J. Phys..

[3] Chen H, Chan CT (2007). Acoustic cloaking in three dimensions using acoustic metamaterials. Appl. Phys. Lett..

[4] Norris AN (2008). Acoustic cloaking theory. Proc. R. Soc. A Math. Phys. Eng. Sci..

[5] Farhat M (2008). A homogenization route towards square cylindrical acoustic cloaks. N. J. Phys..

[6] Torrent D, Sánchez-Dehesa J (2008). Acoustic cloaking in two dimensions: A feasible approach. N. J. Phys..

[7] Zhang S, Xia C, Fang N (2011). Broadband acoustic cloak for ultrasound waves. Phys. Rev. Lett..

[8] Popa B-I, Zigoneanu L, Cummer SA (2011). Experimental acoustic ground cloak in air. Phys. Rev. Lett..

[9] Zigoneanu L, Popa B-I, Cummer SA (2014). Three-dimensional broadband omnidirectional acoustic ground cloak. Nat. Mater..

[10] Kan W (2016). Three-dimensional broadband acoustic illusion cloak for sound-hard boundaries of curved geometry. Sci. Rep..

[11] Alù A, Engheta N (2005). Achieving transparency with plasmonic and metamaterial coatings. Phys. Rev. E.

[12] Guild MD, Alù A, Haberman MR (2011). Cancellation of acoustic scattering from an elastic sphere. J. Acoust. Soc. Am..

[13] Guild MD, Haberman MR, Alù A (2012). Plasmonic-type acoustic cloak made of a bilaminate shell. Phys. Rev. B.

[14] Ammari H, Kang H, Lee H, Lim M (2013). Enhancement of near-cloaking. Part II. The Helmholtz equation. Commun. Math. Phys..

[15] Wang X, Semouchkina E (2013). A route for efficient non-resonance cloaking by using multilayer dielectric coating. Appl. Phys. Lett..

[16] Mirzaei A, Miroshnichenko AE, Shadrivov IV, Kivshar YS (2015). All-dielectric multilayer cylindrical structures for invisibility cloaking. Sci. Rep..

[17] Serna A, Molina LJ, Rivero J, Landesa L, Taboada JM (2018). Multilayer homogeneous dielectric filler for electromagnetic invisibility. Sci. Rep..

[18] Farhat M, Guenneau S, Alù A, Wu Y (2020). Scattering cancellation technique for acoustic spinning objects. Phys. Rev. B.

[19] García-Chocano VM (2011). Acoustic cloak for airborne sound by inverse design. Appl. Phys. Lett..

[20] Andkjær J, Sigmund O (2011). Topology optimized low-contrast all-dielectric optical cloak. Appl. Phys. Lett..

[21] Lan L, Sun F, Liu Y, Ong CK, Ma Y (2013). Experimentally demonstrated a unidirectional electromagnetic cloak designed by topology optimization. Appl. Phys. Lett..

[22] Yamada T, Watanabe H, Fujii G, Matsumoto T (2013). Topology optimization for a dielectric optical cloak based on an exact level set approach. IEEE Trans. Magn..

[23] Fujii G, Watanabe H, Yamada T, Ueta T, Mizuno M (2013). Level set based topology optimization for optical cloaks. Appl. Phys. Lett..

[24] Fujii G, Ueta T (2016). Topology-optimized carpet cloaks based on a level-set boundary expression. Phys. Rev. E.

[25] Nakamoto K, Isakari H, Takahashi T, Matsumoto T (2017). A level-set-based topology optimisation of carpet cloaking devices with the boundary element method. Mech. Eng. J..

[26] Kishimoto N, Izui K, Nishiwaki S, Yamada T (2017). Optimal design of electromagnetic cloaks with multiple dielectric materials by topology optimization. Appl. Phys. Lett..

[27] Fujii G, Takahashi M, Akimoto Y (2021). Acoustic cloak designed by topology optimization for acoustic-elastic coupled systems. Appl. Phys. Lett..

[28] Jo C, Jeong J, Kwon B-J, Park K-C, Oh I-K (2015). Omnidirectional two-dimensional acoustic cloak by axisymmetric cylindrical lattices. Wave Motion.

[29] Liu Z (2000). Locally resonant sonic materials. Science.

[30] Foldy LL (1945). The multiple scattering of waves. I. General theory of isotropic scattering by randomly distributed scatterers. Phys. Rev..

[31] Lax M (1951). Multiple scattering of waves. Rev. Mod. Phys..

[32] Martin PA (2006). Multiple Scattering: Interaction of Time-harmonic Waves with N Obstacles.

[33] Bousse L, Dijkstra E, Guenat O (1996). High-density arrays of valves and interconnects for liquid switching. Hilton Head.

[34] Kittel C, Holcomb DF (1967). Introduction to solid state physics. Am. J. Phys..

[35] Kraft, D. A software package for sequential quadratic programming. Tech. Rep. 28 (1988).

[36] Kraft D (1994). Algorithm 733: TOMP-Fortran modules for optimal control calculations. ACM Trans. Math. Softw. (TOMS).

[37] Johnson, S. G. The NLopt nonlinear-optimization package.

[38] Astley RJ (1996). FE mode-matching schemes for the exterior Helmholtz problem and their relationship to the FE-DtN approach. Commun. Numer. Methods Eng..

[39] Hecht F (2012). New development in freefem++. J. Numer. Math..

[40] Henríquez VC, García-Chocano VM, Sánchez-Dehesa J (2017). Viscothermal losses in double-negative acoustic metamaterials. Phys. Rev. Appl..

